# Nuclear Imaging for Bone Metastases in Prostate Cancer: The Emergence of Modern Techniques Using Novel Radiotracers

**DOI:** 10.3390/diagnostics11010117

**Published:** 2021-01-13

**Authors:** Wietske I. Luining, Dennie Meijer, Max R. Dahele, André N. Vis, Daniela E. Oprea-Lager

**Affiliations:** 1 Department of Urology, Prostate Cancer Network Netherlands, Amsterdam University Medical Center, VU University, 1081 HV Amsterdam, The Netherlands; d.meijer2@amsterdamumc.nl (D.M.); a.vis@amsterdamumc.nl (A.N.V.); 2 Department of Radiology & Nuclear Medicine, Cancer Center Amsterdam, Amsterdam University Medical Center, VU University, 1081 HV Amsterdam, The Netherlands; d.oprea-lager@amsterdamumc.nl; 3 Department of Radiation Oncology, Cancer Center Amsterdam, Amsterdam University Medical Center, VU University Amsterdam, 1081 HV Amsterdam, The Netherlands; m.dahele@amsterdamumc.nl

**Keywords:** prostate cancer, bone metastases, bone scintigraphy, conventional imaging, PET/CT

## Abstract

Accurate staging of prostate cancer (PCa) at initial diagnosis and at biochemical recurrence is important to determine prognosis and the optimal treatment strategy. To date, treatment of metastatic PCa has mostly been based on the results of conventional imaging with abdominopelvic computed tomography (CT) and bone scintigraphy. However, these investigations have limited sensitivity and specificity which impairs their ability to accurately identify and quantify the true extent of active disease. Modern imaging modalities, such as those based on the detection of radioactively labeled tracers with combined positron emission tomography/computed tomography (PET/CT) scanning have been developed specifically for the detection of PCa. Novel radiotracers include ^18^F-sodium fluoride (NaF), ^11^C-/^18^F-fluorocholine (FCH), ^18^F-fluordihydrotestosterone (FDHT), ^68^Gallium and ^18^F-radiolabeled prostate-specific membrane antigen (e.g., ^68^Ga-PSMA-11, ^18^F-DCFPyL). PET/CT with these tracers outperforms conventional imaging. As a result of this, although their impact on outcome needs to be better defined in appropriate clinical trials, techniques like prostate-specific membrane antigen (PSMA) PET/CT have been rapidly adopted into clinical practice for (re)staging PCa. This review focuses on nuclear imaging for PCa bone metastases, summarizing the literature on conventional imaging (focusing on CT and bone scintigraphy—magnetic resonance imaging is not addressed in this review), highlighting the prognostic importance of high and low volume metastatic disease which serves as a driver for the development of better imaging techniques, and finally discussing modern nuclear imaging with novel radiotracers.

## 1. Introduction

Prostate cancer (PCa) is the second-most commonly diagnosed cancer in men worldwide, and has the highest incidence of all cancers among men in the Western world. In 2018, there were an estimated 1.3 million new cases and 359,000 deaths from PCa globally [1,2,3]. The behavior of PCa varies widely, from indolent to highly aggressive. In routine practice, initial clinical suspicion of PCa is usually triggered by an elevated prostate-specific antigen (PSA) and/or an abnormal digital rectal examination (DRE). For a definitive diagnosis, histopathological confirmation is required, and typically obtained by transrectal ultrasound (TRUS) guided needle biopsies [4]. PCa has been classified into five prognostically distinct Grade Groups (GGs) by the International Society of Urological Pathology (ISUP), based on the Gleason Score (GS) [5].

The European Association of Urology (EAU) risk classification [4] (based on the D’Amico classification including initial PSA-value, clinical T-stage and biopsy GG [6]) is commonly used as a prognostic parameter to predict the risk of recurrence, dividing patients into three categories (low, intermediate and high-risk). Patients with high-risk, locally-advanced PCa have an increased risk for the development of metastases, and disease recurrence [4]. The most frequent sites of distant metastases are lymph nodes outside the pelvis (M1a) and bone (M1b) with occasional metastases elsewhere (e.g., visceral organs) (M1c).

Accurate staging of PCa, both at initial diagnosis and at biochemical recurrence (BCR) after previous curative-intent therapy, is important to determine prognosis, and for selecting the optimal treatment strategy. According to the current EAU guidelines, metastatic screening by means of “at least” an abdominopelvic computed tomography (CT)-scan and bone scintigraphy (BS) (^99m^Tc-phosphonate), is recommended in patients with intermediate or high-risk PCa to evaluate the extent of extra-prostatic disease [4]. However, these conventional imaging modalities have limited sensitivity and specificity, affecting their ability to accurately quantify the true extent of disease, especially at low PSA-levels or in the setting of limited volume, oligometastatic disease. This has led to an ongoing search for better imaging tests. As a result of this, prostate-specific membrane antigen (PSMA) positron emission tomography (PET/CT), has been recently introduced. This modern imaging technique using a novel radiotracer has shown high-levels of diagnostic accuracy in the detection of metastatic disease [7,8,9,10], and outperforms conventional imaging in primary staging of PCa [8].

This review focusses on bone metastases, beginning with an overview of conventional imaging in the detection and management of PCa bone disease, touching on the prognostic importance of high and low volume metastatic disease, before highlighting the potential for improvement with modern imaging techniques based on novel radiotracers.

## 2. Conventional Imaging in the Detection and Management of Prostate Cancer

### 2.1. Bone Scintigraphy: The Historical Standard for Nuclear Imaging in Prostate Cancer

The ^99m^Technetium BS is the most widely used imaging modality for the identification of PCa bone metastases, especially in the context of primary staging [4]. Sheikhbahaei et al. [11] recently investigated the diagnostic accuracy of different modalities for the detection of bone metastases in PCa. In this meta-analysis, planar BS was found to have a sensitivity and specificity on a per patient basis of 83% (95%CI 74–90) and 62% (95%CI 48–74), respectively. Adding Single-Photon Emission Computed Tomography with or without CT (SPECT ± CT) to bone scanning, the sensitivity and specificity of the combination increased to 87% (95%CI 76–94) and 75% (95%CI 61–85), respectively. All these values were lower when analyzed on a per lesion basis. Another recent meta-analysis showed a comparable high sensitivity and specificity for BS, on a per patient basis of 79% (95%CI 73–83) and 82% (95%CI 78–85), respectively. Again, the diagnostic performance was lower when analyzed on a per lesion basis, namely 59% (95%CI 55–63) and 75% (95%CI 71–79), respectively [12]. Due to its moderate sensitivity (mainly in patient-based analyses and with the addition of SPECT/CT), wide availability, and low cost, BS is the mainstay for skeletal staging in high-risk PCa.

The diagnostic yield of BS is mainly influenced by three prognostic factors: clinical T-stage, PSA-levels, and biopsy GG [13]. The mean BS positivity rate among 23 studies that included only newly diagnosed PCa patients without previous treatment, was 2.3% in patients with a PSA < 10 ng/mL, 5.3% in patients with PSA 10 < 20 ng/mL, and 16.2% in patients with a PSA 20 < 50 ng/mL [14]. The metastasis detection rate in patients with organ-confined (T1 to T2) and locally advanced (T3 to T4) disease, was 6.4% and 49.5%, respectively. Patients with GG 1–3 and GG 4–5 had a metastasis detection rate of 5.6% and 29.9%, respectively. However, the major limitation of the BS is its moderate to low specificity. Technetium-uptake is not tumor specific, making it challenging to distinguish between different pathological bone conditions (e.g., infectious, traumatic, neoplastic or other benign origin), leading to a higher than desirable false-positive rate.

### 2.2. Computed Tomography in the Workup of Prostate Cancer

Abdominopelvic CT scan is largely used in staging PCa for the identification of lymph node involvement, mainly relying on morphology features (size, shape, and internal architecture) [15]. Lymph nodes are classified as malignant if the node is morphologically abnormal, regardless of nodal size, or when the short-axis exceeds a certain threshold, commonly 8 mm in the pelvis, or >10 mm outside the pelvis [4,16]. However, these thresholds are debated, and the size of non-metastatic lymph nodes varies widely, showing overlap with malignant lymph nodes, and vice-versa (with some malignant nodes being much smaller than the above thresholds) [4,15,16,17]. The sensitivity and specificity for detection of malignant lymph nodes on CT is affected by the threshold used, and also by the inability to differentiate between inflammatory/other benign causes of lymphadenopathy and malignant enlargement [17,18,19]. The estimated sensitivity and specificity of CT for the detection of lymph node metastases have been previously described as less than 40%, and 95%, respectively [19,20,21,22]. Gabriele et al. [23] analyzed 1091 patients previously staged with CT, who underwent surgery (prostatectomy with a pelvic lymph node dissection) demonstrating a CT sensitivity and specificity of 8.8% and 98%, respectively. CT is not as good at detecting architectural changes within normal-sized lymph nodes, and surgically detected metastases are often microscopic—too small to be visualized on standard cross-sectional imaging. Additionally, PCa is nowadays more frequently diagnosed at an earlier stage of disease and at relatively low PSA levels, limiting the likelihood of detecting lymph node metastases on CT. Excessive reliance on CT risks underestimating metastatic spread and creates opportunities for the use and development of more accurate, and novel, imaging techniques [19].

## 3. Initial Treatment of Metastatic Prostate Cancer and Importance of High vs. Low Burden of Disease: Rationale for Improved Imaging and Metastasis Detection

The current treatment of metastatic PCa is mainly based on studies using conventional imaging with BS and abdominopelvic CT. The majority of patients with metastatic hormone-sensitive prostate cancer (mHSPCa) initially respond to androgen-deprivation therapy (ADT) which is the cornerstone of systemic treatment for mHSPCa [24]. However, the duration of response and the interval to developing castration-resistant prostate cancer (CRPC) is highly variable. To potentially delay the development of CRPC, and improve overall survival (OS), combinations of ADT with other systemic agents have been investigated extensively. Three large randomized controlled trials compared ADT plus docetaxel, with ADT-alone in mHSPCa patients. All patients had metastatic PCa at diagnosis or had developed metastases after previous treatment for local disease. The primary endpoint of these studies was OS [25,26,27]. For subanalyses, volume of metastatic disease (low or high volume) was assessed using conventional imaging to categorize patients according to the ChemoHormonal Therapy Versus Androgen Ablation Randomized Trial for Extensive Disease in Prostate Cancer (CHAARTED) criteria [27]. High volume disease was defined as the presence of visceral metastases and/or ≥4 bone lesions of which ≥1 outside was the vertebral bodies and pelvis.

The Groupe d’Etudes des Tumeurs Uro-Genitales and Association Française d’Urologie 15 (GETUG 15 study) [25] was the first to assess the addition of docetaxel to ADT in patients with mHSPCa. In total, 385 patients with newly diagnosed PCa were included, with a median follow-up of 50 months. Key inclusion criteria were radiological evidence of metastatic disease and Karnofsky performance score ≥ 70. Patients were stratified based on previous local treatment and Glass risk group (i.e., PSA, GG, performance status (PS), and location of osseous metastases). ADT plus docetaxel treatment did not significantly increase OS compared to ADT-alone (Hazard Ratio (HR) 1.01, 95%CI 0.75–1.36, *p* = 0.955). Serious adverse events (SAE) occurred in 72 patients (38%) in the combined therapy group, and four treatment-related deaths were described. No SAEs were reported in the ADT-alone arm. In 2016, the survival analysis was updated, and patients were stratified into high (48%) or low (52%) volume metastatic disease according to the CHAARTED (ChemoHormonal Therapy Versus Androgen Ablation Randomized Trial for Extensive Disease in Prostate Cancer) criteria [27]. The median OS in patients with high and low volume disease showed no significant improvement when docetaxel was added to ADT (high volume: 39.8 vs. 35.1 months with ADT alone, HR 0.78, 95%CI 0.56–1.09, *p* = 0.14; low volume: HR 1.02, 95%CI 0.67–1.55, *p* = 0.9) [28].

The second study, the CHAARTED trial [27], enrolled 790 patients with a median follow-up of 28.9 months. Key inclusion criteria were radiological evidence of metastatic disease and Eastern Cooperative Oncology Group (ECOG) PS of 0–2. Stratification was performed according to metastatic disease volume (high and low volume disease) based on conventional imaging (e.g., BS and CT). Patients with high volume disease accounted for 66.2% and 63.6% of the total patients in the ADT plus docetaxel and ADT-alone groups respectively. The median OS for the whole study population was 13.6 months longer in the ADT plus docetaxel group compared to ADT-alone (57.6 versus 44.0 months, HR 0.61, 95%CI 0.47–0.80, *p* < 0.001) and 17 months longer in patients with high volume disease (49.2 versus 32.2 months, HR 0.60, 95%CI 0.45–0.81, *p* < 0.001). Patients assigned to ADT plus docetaxel reported any grade 3 and 4 adverse event in 16.7% and 12.6%, respectively. In 2018, a longer median follow-up (53.7 months) confirmed the OS gain when docetaxel was added to ADT. The OS was 10.4 months longer than with ADT-alone (57.6 versus 47.2 months, HR 0.72, 95%CI 0.59–0.89, *p* = 0.0018) for the whole group and 16.8 months in patients with high volume disease (51.2 versus 34.4 months, HR 0.63, 95%CI 0.50–0.79, *p* < 0.001). There was no benefit detected in patients with low volume disease (HR 1.04, 95%CI 0.70–1.55, *p* = 0.86) [29].

The third trial, Systemic Therapy in Advancing or Metastatic Prostate Cancer: Evaluation of Drug Efficacy (STAMPEDE) study [26], a multi-arm multi-stage trial included 2962 patients with a median follow-up of 43 months. There was metastatic disease in 1817 patients (61%). The standard of care group (ADT-alone) comprised 1184 patients. Docetaxel was combined with ADT in two experimental study arms: ADT plus docetaxel (*n* = 592) and ADT, docetaxel and zoledronic acid (*n* = 593). Key inclusion criteria were that patients were scheduled for long-term ADT, patients had newly diagnosed metastatic or node positive PCa, high-risk locally advanced disease, or relapse after local treatment with high-risk features. Imaging for metastases comprised whole-body BS, and CT or magnetic resonance imaging (MRI) scans. The ADT plus docetaxel group had a significant OS benefit compared to the ADT-alone group (81 versus 71 months, HR 0.78, 95%CI 0.66–0.93, *p* = 0.006). The benefit seemed to be greater in patients with metastatic disease (HR 0.76, 95%CI 0.62–0.92, *p* = 0.005). No evidence of OS improvement was found with the addition of zoledronic acid. The incidence of adverse events grade ≥ 3 was 52% in both docetaxel containing arms (versus 32% in the two non-docetaxel containing arms). In the post-hoc analysis of the STAMPEDE trial in 2018, after a median follow-up of 78.2 months, patients were again stratified by volume of metastatic disease using the CHAARTED criteria [27]. This was assessable for 76% (830/1086) of metastatic patients, 44% of whom had low volume and 56% high volume disease. The OS benefit for ADT plus docetaxel compared to ADT-alone was 59.1 versus 43.1 months (HR 0.81, 95%CI 0.69–0.95, *p* = 0.003). The hazard ratios were consistent in the low (HR 0.76, 95%CI 0.54–1.07 *p* = 0.107) and high (0.81, 95%CI 0.64–1.02, *p* = 0.064) volume disease subgroups [30].

## 4. Modern Nuclear Imaging in Prostate Cancer: New PET/CT Radiotracers

The three studies described above were all based on the use of conventional imaging (i.e., BS, CT and/or MRI), upon which the current treatment of metastatic PCa is mainly based. However, PCa imaging is evolving rapidly. Over the last few years, modern PET/CT imaging techniques, using radioactively labelled tracers have been introduced to the diagnostic armamentarium. In clinical practice it is preferable to use PET radiopharmaceuticals with high tumor-specific uptake and low background activity, capable of diagnosing bone, lymph node, and visceral metastases. Various tracers have been developed for metastatic PCa, based on osteoblastic activity (^18^F-sodium fluoride (NaF)), cellular phospholipid membrane proliferation (^11^C-/^18^F-fluorocholine (FCH)), androgen receptor expression (^18^F-fluordihydrotestosterone (FDHT)) and targeting the prostate-specific membrane antigen (^68^Gallium (^68^Ga) or ^18^Flourine (^18^F)) [31] (Table 1).

The ^18^F-NaF PET/CT enables accurate detection of osseous metastases, but is nonspecific for lymph node metastatic disease and is therefore not suitable for comprehensive staging of metastatic PCa. Uptake of ^18^F-NaF is determined by osteoblastic activity as it attaches to sites of new bone formation [31]. A recent meta-analysis showed a pooled sensitivity and specificity of ^18^F-NaF PET/CT for the detection of bone metastases on a per patient basis of 98% (95%CI 95–99) and 90% (95%CI 86–93), and on per a lesion basis of 97% (95%CI 95–98) and 84% (95%CI 81–87), respectively. The diagnostic performance of ^18^F-NaF PET/CT is superior compared to BS [11]. However, in patients with newly diagnosed PCa scheduled for radical prostatectomy, no added value of ^18^F-NaF PET/CT was found for the detection of bone metastases in case of a negative BS [32]. The advantages of ^18^F-NaF PET/CT include: superior image quality, due to a higher bone uptake and faster blood clearance, and superior spatial resolution, with better definition of bone metastases, thus contributing to a higher diagnostic accuracy. However, BS has advantages over ^18^F-NaF PET/CT in terms of cost-effectiveness and availability, and therefore it remains the preferred technique for generalized use.

The relatively new oncological tracer, ^18^F-FDHT, is a radiolabeled analogue of dihydrotestosterone, directly binding to the androgen receptor (AR). It allows in-vivo visualization and quantification of AR expression [33,34]. The AR is crucial for PCa growth, and essential for AR-directed therapies in metastatic CRPC. ^18^F-FDHT PET/CT was successfully used in early phase clinical trials to demonstrate AR specific drug binding [35,36]. Larson et al. studied ^18^F-FDHT PET uptake in seven patients with progressive clinically metastatic PCa. Conventional imaging identified 59 lesions, and 78% of the lesions (46 of 59 lesions) were ^18^F-FDHT positive [18]. Dehdashti et al. [37] enrolled 19 patients with advanced PCa, with biopsy and/or radiologically proven metastatic disease, and found a sensitivity for ^18^F-FDHT PET of 63%. This finding suggests that ^18^F-FDHT PET/CT seems to be a promising predictive biomarker in the evaluation of AR status, and for treatment response assessment, rather than for the primary detection of PCa metastases. Further investigation is needed and it has not yet entered routine clinical use.

PCa cells are known for their increased proliferation and upregulation of choline kinase. Choline is a precursor for the biosynthesis of phosphatidylcholine which is a key component of cell membrane proliferation. This amino acid can be targeted with ^11^C- or ^18^F, resulting in radio-labeled choline. These radiotracers are extensively used in PCa, particularly in the setting of BCR, and as potential biomarkers of response after chemotherapy [38]. Shen et al. [12] found, on a per patient analysis, a pooled sensitivity and specificity for the detection of bone metastases using choline PET/CT in patients with PCa of 87% and 97%, respectively. Radiolabeled choline PET/CT has been shown to have a pooled sensitivity and specificity in recurrent disease for all sites (prostate, lymph nodes, bone) of 85.6% and 92.6%, respectively [39]. A limitation of ^18^F-Choline is the low sensitivity for the detection of PCa metastases (bone and lymph node) at low PSA-values, where it is clearly outperformed by radiolabeled-PSMA [40,41].

PSMA-PET/CT is a novel imaging technique increasingly used in routine practice. PSMA is a class II cell-surface transmembrane protein overexpressed in malignant prostatic epithelial cells, making it an excellent target for imaging. The degree of PSMA-expression is correlated with higher tumor grades, and higher risk of disease progression, leading to it being described as a marker of disease aggressiveness [42,43]. In a recent study of 90 patients with biopsy proven primary PCa, PSA-value and GG correlated with the intensity of tracer expression on ^68^Ga-PSMA-11 PET/CT, with a significantly higher tumor-related tracer uptake seen in patients with either PSA ≥ 10 ng/mL or GG ≥ 4 [44].

**Figure 1:**
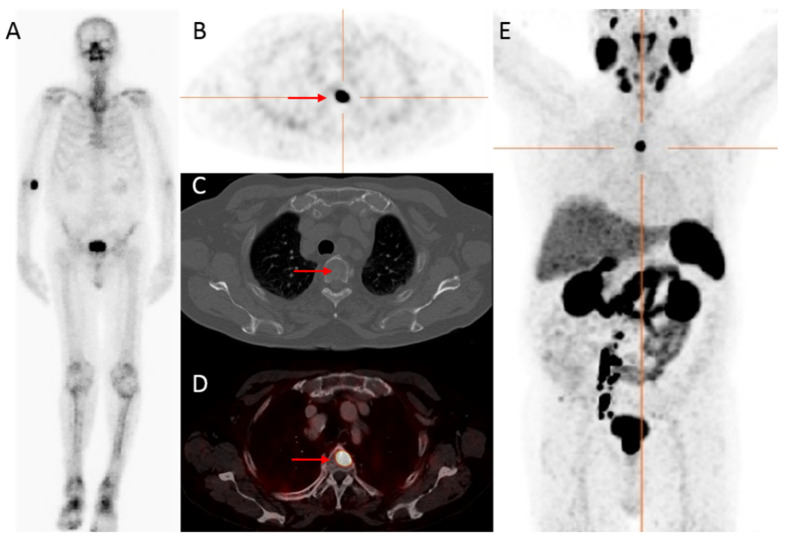
An 83-year-old patient, with castration-resistant prostate cancer (CRPC) after initial treatment with hormonal therapy (2009), and secondary abiraterone (2019), showed improved detection of bone metastatic prostate cancer PCa with ^68^Ga-PSMA PET/CT compared to bone scintigraphy. The prostate—specific antigen (PSA)—value at PET scanning was 25.9 ng/mL. On the bone scintigraphy, no suspect bone metastases were visualized (**A**). Transversal ^68^Ga-PSMA PET (**B**), fused PET/CT (**D**) and maximum intensity projection (MIP) (**E**) revealed a lesion located in the thoracic spine with increased PSMA expression (red arrow), with no evident substrate on CT (**C**). Time interval between bone scintigraphy and ^68^Ga-PSMA PET/CT was 5 weeks.

^68^Ga-labeled PSMA tracers are the most intensively studied, demonstrating high detection rates for both bone and lymph node metastases [7,9,45]. Perera et al. [9] evaluated the diagnostic accuracy of ^68^Ga-PSMA-11 PET/CT for the detection of metastatic disease in both primary (high-risk and advanced prostate cancer) and secondary staging (at BCR) (Figure 1). The lesion-based analysis showed a pooled sensitivity of 75%, and specificity of 99% for primary staging, with corresponding figures of 77% and 97%, respectively, for a per patient analysis. The positivity rate of the ^68^Ga-PSMA PET/CT in secondary staging increased in patients with higher PSA-levels: 33% (PSA < 0.2 ng/mL), 45% (0.2–0.49 ng/mL), 59% (0.5–0.99 ng/mL), 75% (1–1.99 ng/mL), and 95% (≥2 ng/mL). These percentages are substantially higher than those for conventional imaging techniques.

PSMA PET/CT has shown high detection rates (98–100%) for the primary prostate tumor [46,47,48], and provides more sensitive screening for metastatic disease at initial staging than conventional imaging modalities [8]. A recent meta-analysis confirmed the higher diagnostic accuracy of ^68^Ga-PSMA PET/CT compared to BS with higher sensitivity (0.97 versus 0.86) and specificity (1.00 versus 0.95) for detecting bone metastases [10]. Hofman et al. [8] prospectively compared ^68^Ga-PSMA PET/CT with conventional imaging in patients with high-risk PCa (*n* = 302). ^68^Ga-PSMA PET/CT showed an enhanced diagnostic accuracy for identifying either pelvic nodal or distant metastases compared to conventional imaging (*p* < 0.0001). In subgroup analysis, ^68^Ga-PSMA PET/CT was superior in detecting pelvic lymph node metastases (91% versus 59%), and distant metastases (95% versus 74%). First-line ^68^Ga-PSMA PET/CT (*n* = 148) found abdominal lymph node metastases in 13 patients (9%), bone metastases in 15 patients (10.1%), and visceral metastases in one (1%). In the primary staging of PCa, conventional imaging and ^68^Ga-PSMA PET/CT led to a change in treatment approach in 23 (15%) and 41 patients (28%), respectively (*p* = 0.008).

**Figure 2:**
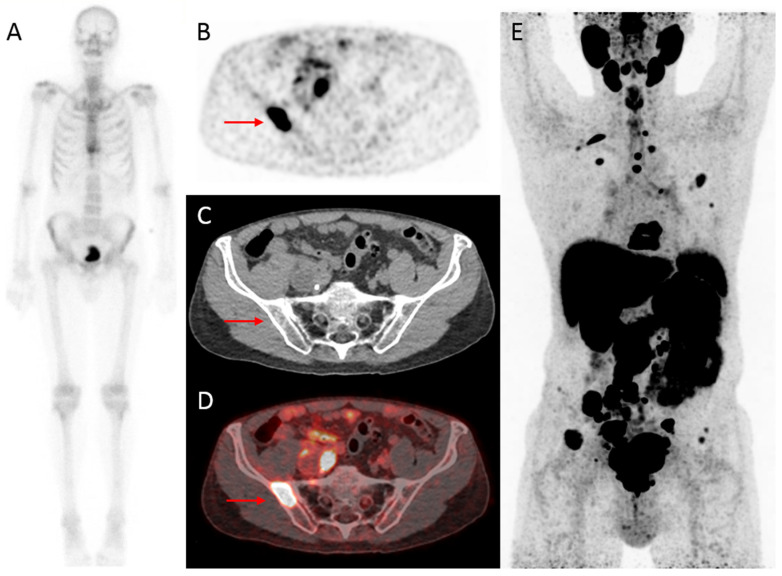
Initial assessment of a 75-year old patient, newly diagnosed with PCa (Grade Group (GG) 5), with an initial PSA-value of 1396 ng/mL. On bone scintigraphy, the increased uptake in the thoracic spine was attributed to an (osteoporotic) collapsed vertebra, and the faint uptake in the left third rib to a post-traumatic origin. Despite the high PSA-value, no abnormal uptake consistent with osseous metastases was visualized (**A**). However, extensive metastatic disease was found on ^18^F-DCFPyL PET/CT (**B**–**E**). For example, transversal ^18^F-DCFPyL PET (**B**) and fused PET/CT (**D**) showed highly increased PSMA-expression in the right iliac bone (red arrow, maximum standardized uptake value (SUV_max_) 8.15), compatible with a lytic lesion on CT (**C**). The time interval between bone scintigraphy and ^18^F-DCFPyL PET/CT was 5 days.

Next to the intensively studied ^68^Ga-labeled PSMA tracers, ^18^F-labeled tracers, such as ^18^F-DCFPyL [49] and ^18^F-PSMA-1007 [50], appear promising. ^18^F-labeled tracers are attractive due to a shorter positron range and higher positron yield compared to ^68^Ga, providing higher resolution PET-images which may improve early detection of small metastases [45], and ^18^F-DCFPyL has shown higher tumor to background ratios compared to ^68^Ga-PSMA [51]. For ^18^F-DCFPyL, initial experience with the detection of bone metastases in primary prostate cancer has been published (Figure 2), but the diagnostic accuracy of ^18^F-DCFPyL PET/CT for detecting pelvic lymph nodes metastases in initial staging is less well described. Wondergem et al. [52] enrolled 160 patients with high-risk PCa who underwent an ^18^F-DCFPyL PET/CT for primary staging. PSMA-positive bone metastases were detected in 49 patients (31%) and lymph nodes were found in 81 patients (51%) of which 52% (*n* = 42) were enlarged on CT-scan. The treatment plan was adjusted after ^18^F-DCFPyL in 17% (*n* = 27) of the patients. Jansen et al. [53] prospectively analyzed 117 patients who underwent imaging prior to robot-assisted radical prostatectomy and extended pelvic lymph node dissection, in order to determine the diagnostic accuracy of ^18^F-DCFPyL PET/CT for pelvic lymph node staging in intermediate- (36.8%) and high-risk (63.3%) PCa. Histological lymph node metastases were found in 17/117 (14.5%) patients, of whom seven had a suspicious PET/CT, resulting in a limited sensitivity (41.2%), but high specificity (94.0%). The low sensitivity is explained by the median tumor size of 1.5 mm for PET/CT undetected lymph node metastases and illustrates the “resolution” challenges faced by the newly developed imaging techniques.

With the better performance of novel imaging modalities, patients with BCR can be diagnosed earlier with metastatic disease. Consequently, the number of patients diagnosed with oligometastatic PCa (usually considered to be a maximum of 3–5 metastases) has increased. The true oligometastatic state is considered to be one of limited metastatic potential in which the local treatment of all visible metastases has the potential to bring about long-term survival in some patients [54]. The treatment of oligometastatic disease (in practice mostly patients with 1–2 lesions) now attracts considerable interest [55]. The most studied treatment approach in patients with oligometastatic recurrent PCa, is metastasis-directed local therapy (MDT; for example, stereotactic radiotherapy or surgery), with the potential goals of delaying the start of ADT and influencing prognosis [56]. A recent randomized phase II study of patients with oligometastatic recurrent PCa showed improved ADT-free survival in patients who underwent MDT compared to surveillance [57]. The effect of MDT on OS needs to be addressed in further clinical trials.

In summary, PSMA PET/CT has the potential for more accurate metastasis detection and (re)staging at initial diagnosis and at BCR than standard conventional imaging modalities (in practice BS and CT). This could potentially lead to changes in treatment and selection of more optimal strategies. However, the clinical benefit of (even) earlier detection of metastases has yet to be shown in well-powered randomized studies. As a result, current EAU guidelines do not recommend the routine use of PSMA (PET/CT) in their imaging algorithms [4]. Currently, at Amsterdam UMC, location VUmc, we are conducting two prospective, clinical trials looking at the diagnostic accuracy of ^18^F-DCFPyL PET/CT compared to conventional imaging for the detection of metastases in patients with newly diagnosed high risk PCa who have a negative BS (trial 1, VUmc IRB number: 2019.051) or a positive BS with low volume disease (trial 2, VUmc IRB number: 2019.054). The change in treatment approach will be evaluated as a secondary outcome.

## 5. Conclusions

Accurate staging of PCa both at initial diagnosis and at BCR is important to determine prognosis, and for optimal treatment selection. Although current guidelines recommend “at least” conventional imaging (e.g., BS and CT) for metastatic screening in intermediate and high risk patients, it has limited sensitivity and specificity for the detection of metastases compared to modern imaging modalities, such as PSMA PET/CT. This has led to the rapid adoption of techniques like PSMA PET/CT into routine clinical practice. Further prospective trials are warranted to investigate whether this enhanced detection actually leads to improved oncological outcomes to quantify the gains that can be expected from new imaging techniques and to confirm their place in the diagnostic hierarchy.

## Figures and Tables

**Table 1:** Diagnostic performance of selected imaging methods for the detection of prostate cancer bone metastases.

	Sensitivity (%)	Specificity (%)	Reference	Type of Article
Bone scintigraphy	79	82	Shen [12]	Meta-analysis
CT	8.8	98	Gabriele [23]	Retrospective cohort
^18^F-NaF PET/CT	98	90	Sheikhbahaei [11]	Meta-analysis
^18^F-FDHT PET/CT	63	-	Dehdashti [37]	Prospective cohort
^18^F-FCH PET/CT	87	97	Shen [12]	Meta-analysis
^68^Ga-PSMA PET/CT	77	97	Perera [9]	Systematic review and meta-analysis
^18^F-DCFPyL PET/CT	-	-	-	-

CT: computed tomography; PET: positron emission tomography; ^18^F: ^18^Flourine; NaF: sodium fluoride; FDHT: fluorodihydrotestosterone; FCH: ^f^luorocholine; ^68^Ga-PSMA: ^68^Gallium prostate-specific membrane antigen; DCFPyL: (2-(3-{1-carboxy-5-[(6-18F-fluoro-pyridine-3-carbonyl)-amino]-pentyl}-ureido)-pentanedioic acid).

## Data Availability

No new data were created or analyzed in this study. Data sharing is not applicable to this article.

## Abbreviations

^68^Ga	^68^Gallium
^18^F	^18^Fluorine
^18^F-FCH	^18^F-fluorocholine
^18^F-FDHT	^18^F-fluordihydrotestosterone
^18^F-NaF	^18^F-sodium fluoride
ADT	androgen-deprivation therapy
AR	androgen receptor
BCR	biochemical recurrence
BS	bone scintigraphy
CRPC	castration-resistant prostate cancer
CT	computed tomography
DRE	digital rectal examination
EAU	European Association of Urology
ECOG	Eastern Cooperative Oncology Group
GG	Grade Group
GS	Gleason Score
HR	hazard ratio
ISUP	International Society of Urological Pathology
mHSPCa	metastatic hormone-sensitive prostate cancer
MRI	magnetic resonance imaging
OS	overall survival
PCa	prostate cancer
PET	positron emission tomography
PS	performance status
PSA	prostate-specific antigen
SAE	serious adverse events
TRUS	transrectal ultrasound
